# The MHC-II transactivator CIITA, a restriction factor against oncogenic HTLV-1 and HTLV-2 retroviruses: similarities and differences in the inhibition of Tax-1 and Tax-2 viral transactivators

**DOI:** 10.3389/fmicb.2013.00234

**Published:** 2013-08-22

**Authors:** Greta Forlani, Rawan Abdallah, Roberto S. Accolla, Giovanna Tosi

**Affiliations:** Laboratory of General Pathology and Immunology, Department of Surgical and Morphological Sciences, University of InsubriaVarese, Italy

**Keywords:** restriction factors, CIITA, HTLV-1 Tax-1, HTLV-2 Tax-2, viral replication

## Abstract

The activation of CD4^+^ T helper cells is strictly dependent on the presentation of antigenic peptides by MHC class II (MHC-II) molecules. MHC-II expression is primarily regulated at the transcriptional level by the *AIR-1* gene product CIITA (class II transactivator). Thus, CIITA plays a pivotal role in the triggering of the adaptive immune response against pathogens. Besides this well known function, we recently found that CIITA acts as an endogenous restriction factor against HTLV-1 (human T cell lymphotropic virus type 1) and HTLV-2 oncogenic retroviruses by targeting their viral transactivators Tax-1 and Tax-2, respectively. Here we review our findings on CIITA-mediated inhibition of viral replication and discuss similarities and differences in the molecular mechanisms by which CIITA specifically counteracts the function of Tax-1 and Tax-2 molecules. The dual function of CIITA as a key regulator of adaptive and intrinsic immunity represents a rather unique example of adaptation of host-derived factors against pathogen infections during evolution.

## INTRODUCTION

Adaptive and innate immune responses represent the most powerful tool used by the host to counteract infectious agents. Additional intrinsic defense systems against viral infections have been recently identified. They include host-encoded restriction factors, initially described for their inhibitory effect on immunodeficiency virus type 1 (HIV-1) infection (reviewed in Wolf and Goff, 2008), such as apolipoprotein B mRNA-editing catalytic polypeptides (APOBECs; Sheehy et al., 2002; Chiu and Greene, 2008; Refsland and Harris, 2013), TRIM (tripartite motif) family members (Stremlau et al., 2004; Ozato et al., 2008; Fletcher and Towers, 2013), tetherin (Neil et al., 2008; Kuhl et al., 2011), and sterile alpha motif (SAM) and HD domain-containing protein 1 (SAMHD1) (Laguette et al., 2011; Sharkey, 2013). Most of these anti-viral proteins were uncovered through the discovery of viral factors that counteract their function, implying that viruses are resistant to the restriction factors of their natural hosts. Although these findings suggested a cross-species restriction, further studies demonstrated that restriction factors may limit pathogenicity *in vivo* even in their specific host (reviewed in Ross, 2009). Besides HIV, the phenomenon of viral restriction has been investigated in other viral infections including oncogenic human T cell lymphotropic virus type 1 (HTLV-1) infection. HTLV-1 was the first human oncogenic retrovirus to be discovered (Poiesz et al., 1980). HTLV-1 is closely related to the less pathogenic HTLV-2 virus. The genomes of these viruses code for similar structural, enzymatic, and regulatory proteins (Franchini, 1995; Nicot et al., 2005). Among them, the transcriptional activators, named Tax-1 (HTLV-1) and Tax-2 (HTLV-2) share roughly 77% amino acid sequence homology and have conserved functional regions. Both viruses infect primarily T lymphocytes, but their infection is associated with different disease manifestations. HTLV-1 is the etiologic agent of an aggressive form of adult T cell leukemia/lymphoma (ATLL), of a neurological disorder designated HTLV-1-associated myelopathy/tropical spastic paraparesis (HAM/TSP) and inflammatory disorders (Yoshida et al., 1982; Uchiyama, 1997; Mahieux and Gessain, 2003). Tax-1 plays a major role in the onset of leukemogenesis by regulating cell cycle progression, cell growth, apoptosis, and DNA repair (Feuer and Green, 2005; Hall and Fujii, 2005; Kashanchi and Brady, 2005; Matsuoka and Jeang, 2007; Yasunaga and Matsuoka, 2011). HTLV-2 has been linked to HAM/TSP “like” cases, whereas no clear epidemiological link to lymphoproliferative malignancies has been demonstrated (Lehky et al., 1996; Roucoux and Murphy, 2004). Comparative studies of Tax-1 and Tax-2 functions brought to light major phenotypic differences in their viral transactivating capacity, transforming activity, modulation of cellular genes expression, and subcellular localization (Semmes et al., 1996; Tanaka et al., 1996; Endo et al., 2002; Sieburg et al., 2004; Shoji et al., 2009; Bertazzoni et al., 2011; Rende et al., 2012; Turci et al., 2012).

Studies on the role of restriction factors in HTLV-1 infection are controversial. HTLV-1 replicates in the same cells as HIV-1 and it does not express an accessory protein analogous to HIV-1 Vif that inactivates hAPOBEC3G. Nevertheless, HTLV-1 seems to be relatively resistant to hAPOBEC3 proteins (Mahieux et al., 2005; Ohsugi and Koito, 2007). Derse et al. (2007) have shown that resistance of HTLV-1 to hAPOBEC3G is mediated by the C-terminus of gag, which seems to exclude hAPOBEC3G from virions. Other reports have shown that hAPOBEC3G is packaged into HTLV-1 particles, but with opposite effects on virion infectivity (Navarro et al., 2005; Sasada et al., 2005). Interestingly, it has been hypothesized that non-sense mutations in viral genes induced by hAPOBEC3G might allow the virus to escape the host immune response (Fan et al., 2010). Studies related to a possible effect of tetherin on HTLV-1 infectivity indicated that tetherin reduces cell-free infectivity of HTLV-1 with a minor effect on cell-to-cell transmission (Ilinskaya et al., 2013). Finally, evidence of HTLV-1 resistance to SAMHD1-mediated restriction have been recently reported (Gramberg et al., 2013).

Another cellular protein with anti-viral function is the MHC class II (MHC-II) transactivator, also designated CIITA (class II transactivator). The gene encoding CIITA and the elucidation of its function as the master regulator of MHC-II gene transcription and, thus, of antigen presentation to CD4^+^ T helper cells (TH) were first discovered in our laboratory (Accolla et al., 1986). Upon antigen recognition TH cells coordinate both humoral and cellular immune responses to eradicate pathogen infections and fight tumors (Accolla and Tosi, 2012). This prominent role of CIITA in the homeostasis of the immune system has emerged from the elucidation of the molecular defect at the basis of the bare lymphocyte syndrome (BLS), a severe form of combined immunodeficiency, characterized by the loss of expression of MHC-II molecules (Yang et al., 1988; Steimle et al., 1993; Reith and Mach, 2001). CIITA is a protein of 1130 amino acids localized in both the nucleus and the cytoplasm; it contains four functional domains: the N-terminal transcription activation domain (AD); the proline/serine/threonine-rich region (P/S/T); the GTP-binding domain (GBD), and the C-terminal leucine-rich repeats (LRR) that are critical for the subcellular distribution of the protein (Cressman et al., 2001). The integrity of CIITA domains is critical for the activation function on the MHC-II promoter. CIITA regulates MHC-II gene expression by coordinating sequential steps of the transcription process from the assembly of the general transcriptional machinery and the recruitment of coactivators and chromatin remodeling factors, to the binding of transcription elongation factors (Fontes et al., 1999a). CIITA is recruited to MHC-II promoters via the interaction with DNA-bound factors including the regulatory factor X (RFX) complex and the trimeric NF-Y complex (Caretti et al., 2000; De Sandro et al., 2000; Masternak et al., 2000; Zhu et al., 2000; Jabrane-Ferrat et al., 2002, 2003). Both constitutive and IFNγ-inducible expression of MHC-II is controlled by CIITA, whose gene is regulated at transcriptional level by three distinct promoters driving CIITA expression in different cell lineages (Reith et al., 2005).

Several years ago, we discovered that CIITA restricts HIV-1 infection by acting at the level of viral replication. The molecular mechanism at the basis of this inhibition is the competition between CIITA and HIV-1 Tat transactivator for the cyclin T1 subunit of the elongation complex P-TEFb (positive transcription elongation factor b; Accolla et al., 2002). In this review we summarize our knowledge of the role of CIITA as a restriction factor for HTLV-1 and HTLV-2 viruses. We discuss the results on the inhibition of Tax-1 and Tax-2 functions by CIITA and concentrate on novel insights into the mechanisms through which CIITA operates this suppressive function.

## CIITA INHIBITS BOTH HTLV-1 AND HTLV-2 VIRAL REPLICATION

**FIGURE 1:**
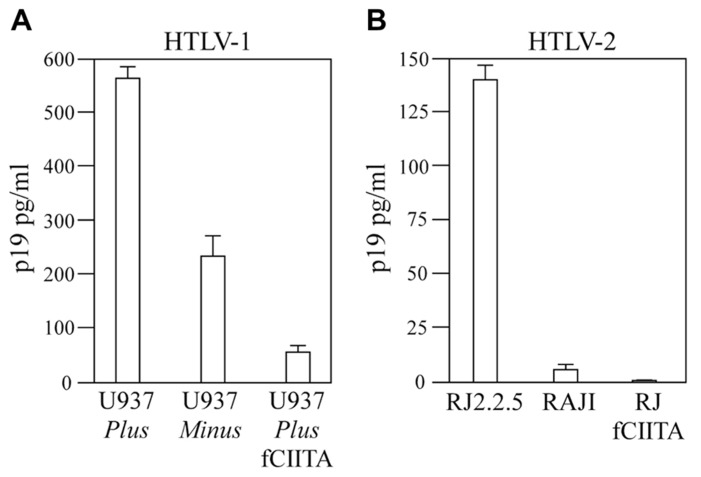
**Endogenous CIITA inhibits both HTLV-1 and HTLV-2 gene expression.** **(A)** CIITA-negative U937 *plus* clone, CIITA-positive U937 *minus* clone, and U937 *plus* clone stably expressing fCIITA (U937 *plus* fCIITA) were transfected with the pACH plasmid containing the entire HTLV-1 genome. The amount of HTLV-1 p19 antigen (pg/ml) in cells supernatants was detected by enzyme-linked immunoassay (ELISA) 48 h post-transfection. Error bars indicate standard deviations. Derived from Tosi et al. (2011). **(B)** CIITA-positive Raji cells, their CIITA-negative isogenic mutant RJ2.2.5, and the RJfCIITA cells stably transfected with CIITA were infected with the HTLV-2 Gu strain 2b and the productive infection was evaluated by the presence of HTLV-2 p19 antigen (pg/ml) in cell culture supernatants measured by (ELISA). Error bars indicate standard deviations. Derived from Casoli et al. (2004).

Beside inhibiting HIV-1 transcriptional elongation, CIITA inhibits also the replication of HTLV-1 and HTLV-2. As far as HTLV-1, we demonstrated that exogenously expressed CIITA in 293T cells transfected with the HTLV-1 molecular clone pACH resulted in strong inhibition of HTLV-1 virus production. More importantly, in promonocytic cells endogenously expressed CIITA produced the same effect (Tosi et al., 2011). Two phenotypically and functionally distinct clones of the promonocytic U937 cell line, named *Minus* and *Plus, *previously characterized for their inefficient or efficient capacity to support productive HIV-1 infection, respectively (Franzoso et al., 1994), were studied. Interestingly, we found that the *Minus* clone expresses CIITA and MHC-II, whereas the *Plus* clone does not express either. *Minus* and* Plus* clones were transfected with the pACH plasmid and assessed for viral expression by measuring p19 antigen in the cell supernatants. Remarkably, and similarly to what observed with the HIV-1 infection, we found that the two clones had a different behavior with respect to HTLV-1 infection. The p19 levels were drastically reduced in the supernatants of CIITA-positive *Minus* clone, compared with the CIITA-negative *Plus* clone. Moreover, the stable expression of CIITA in the *Plus* clone after transfection with CIITA cDNA, reverted its permissive phenotype to the *Minus*-like non-permissive one (**Figure 1A**), demonstrating that CIITA is a major restriction factor for HTLV-1. As far as HTLV-2 infection, we found that cells of both the T- and B-lineage are less permissive to HTLV-2 replication in the presence of CIITA. In particular, by using the isogenic B cell system, consisting of CIITA-positive Raji cells and its CIITA-negative derivative RJ.2.2.5 (Accolla, 1983), it was found that RJ.2.2.5 sustained very high levels of virus replication, whereas a profound inhibition of viral replication was observed in Raji parental cells, although both cell lines were equally infected by HTLV-2. Thus, physiologic levels of CIITA were able to strongly inhibit HTLV-2 expression. Consistent with this observation, the permissive RJ2.2.5 cells stably transfected with CIITA cDNA became refractory to HTLV-2 replication showing almost undetectable levels of p19 antigen upon viral infection (Casoli et al., 2004, **Figure 1B**).

Overall, our observations indicated that physiologic amounts of CIITA may inhibit viral expression in cells that are natural target of HTLV-1 and HTLV-2 infection, suggesting that *in vivo* the virus may replicate preferentially in cells lacking CIITA. In this regard, it is interesting that during dendritic cell (DC) maturation induced by different stimuli (LPS, CD40L, Sendai virus, *Salmonella* typhimurium, IFNα, and TNFα), the expression of MHC-II molecules is increased due to an enhanced transport of preformed molecules to the cell surface. In contrast*, de novo* biosynthesis of MHC-II mRNA is shut off because of the epigenetic silencing of CIITA gene (Landmann et al., 2001). Thus, it is conceivable that a similar CIITA silencing might occur in DC infected by HTLV-1 allowing viral replication and spreading to CD4^+^ T cells (Jones et al., 2008).

## CIITA TARGETS Tax-1 AND Tax-2 TRANSACTIVATORS TO INHIBIT VIRAL EXPRESSION

**FIGURE 2:**
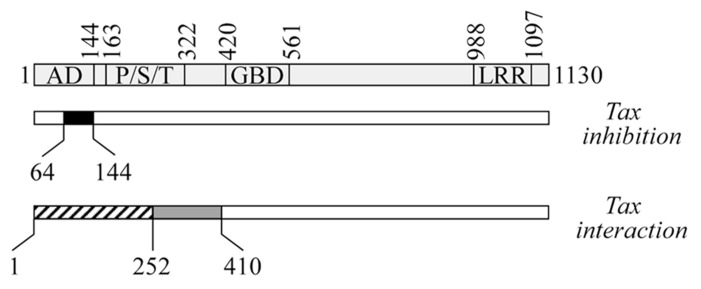
**Schematic representation of CIITA regions involved in the suppression ofTax function and in CIITA-Tax association.** At the top is a diagram of CIITA with its domains: AD, activation domain; P/S/T, proline/serine/threonine-rich domain; GBD, GTP-binding domain; and LRR, leucine-rich repeats. The black box represents the minimal domain from positions 64–144 that is necessary to block the transcription function of Tax (*Tax-inhibition*, middle). Hatched (positions 1–252) and gray (positions 253–410) boxes represent the two regions of CIITA interacting with Tax (*Tax interaction*, bottom).

In searching for the molecular mechanisms through which CIITA inhibits HTLV-1 and HTLV-2 viral replication, we found that CIITA targets the viral transactivators Tax-1 and Tax-2. Indeed, we showed that exogenous CIITA could inhibit the Tax-1- and Tax-2-mediated HTLV LTR transactivation in LTR-driven luciferase gene reporter assays (Casoli et al., 2004; Tosi et al., 2006, 2011; Orlandi et al., 2011). By using several CIITA deletion mutants, the N-terminal region 64–144 was found to be minimally necessary to inhibit both Tax-1 and Tax-2 function (**Figure 2**).

Accordingly, HTLV-2 replication was found to be strongly suppressed in RJ.2.2.5 B cells stably transfected with the N-terminal 1–321 fragment of CIITA, which localizes mostly in the nucleus. In contrast, the cytoplasmic mutant CIITA 322–1130, which does not contain the minimal inhibitory domain, did not significantly inhibit HTLV-2 expression (Tosi et al., 2006).

Similarly, cytoplasmic CIITA mutants containing the region 64–144 and partially and /or temporarily accumulating in the nucleus, inhibited Tax-1 transactivation. This was observed with a mutant having the internal deletion of the region 253–410 mediating dimerization of CIITA (Tosi et al., 2002) and accumulating in the nucleus after treatment with Leptomycin B (LMB), an inhibitor of CRM1-mediated nuclear export. Importantly, the study of this mutant in relation to Tax-1 inhibition, provided new insights on the cellular biology of CIITA. For the first time, phosphorylation-dependent dimerization of CIITA has been defined as a critical post-translational modification (PTM) required for CIITA nuclear retention and, thus, transcriptional activity on MHC-II promoters (Tosi et al., 2011). However, because full length CIITA has a dual nuclear and cytoplasmic localization, our findings do not exclude that CIITA might also inhibit Tax in the cytoplasm. Studies are in progress to assess whether cytoplasmic CIITA mutants containing the N-terminal inhibitory region, still inhibit Tax. These experiments will clarify whether CIITA exerts its suppressive function on Tax in both the nucleus and the cytoplasm, potentially revealing a more complex picture, as CIITA might exploit distinct molecular mechanisms to inhibit Tax in the two cellular compartments.

This will be particularly relevant for Tax-2, which, differently from Tax-1, exhibits a predominant cytoplasmic distribution with some accumulation in nuclear bodies (Meertens et al., 2004a; Sheehy et al., 2006; Turci et al., 2009).

A relevant finding of our studies, instrumental in understanding the complex picture of the CIITA-mediated inhibition of Tax-1 and Tax-2 function, was the demonstration of the *in vivo* molecular interaction between CIITA and Tax-1/Tax-2 (Orlandi et al., 2011; Tosi et al., 2011). Since CIITA is localized predominantly in the nucleus but also to a lesser extent in the cytoplasm, one possible scenario is that the cytoplasmic fraction of CIITA could bind Tax itself or a cellular factor crucial for Tax transactivation, inhibiting their nuclear translocation. Intriguingly, our interaction studies revealed that CIITA associates *in vivo* with Tax-1 and Tax-2 by using two adjacent regions at the N-terminus (**Figure 2**). The region 1–252 mediates both the binding to the transactivators and their functional inhibition, whereas the region 253–410 binds to, but does not inhibit Tax proteins (Orlandi et al., 2011; Tosi et al., 2011). We suggested that the two regions form a single Tax-interacting surface in the context of the entire CIITA molecule, but only the presence of the minimal domain 64–144 confers inhibitory properties to this association. Tax-1 region involved in this interaction spans amino acids sequence 1 to 108, including the CREB (cAMP response element-binding protein)-binding domain (Adya and Giam, 1995; Goren et al., 1995; Tosi et al., 2011). In searching for similar or distinct mechanisms of CIITA-mediated inhibition of Tax-1 and Tax-2, future experiments will assess whether CIITA binds the same N-terminal region of Tax-2. Of note, CIITA-Tax interaction was not observed with proteins produced *in vitro* (data not shown) indicating that a bridging cell factor might play a role in this interaction or that PTM, that do not occur *in vitro*, are crucial to promote the binding. Many studies have shown that sumoylation, ubiquitination, acetylation, and phosphorylation play a critical role in the subcellular localization, protein–protein interaction and function of both Tax-1 and Tax-2, revealing similarities and differences between the two transactivators (Bex et al., 1999; Chiari et al., 2004; Lamsoul et al., 2005; Durkin et al., 2006; Nasr et al., 2006; Gatza et al., 2007; Lodewick et al., 2009; Turci et al., 2009; Bidoia et al., 2010; Bertazzoni et al., 2011; Journo et al., 2013). In addition, dimerization of Tax-1 is necessary for its nuclear localization and interaction with CREB and the 21-bp repeat elements (Tie et al., 1996; Jin and Jeang, 1997; Basbous et al., 2003; Fryrear et al., 2009). Similarly, we previously described that CIITA expressed in cells, but not CIITA produced *in vitro*, forms homodimers in a phosphorylation-dependent manner (Tosi et al., 2002) and this modification is a prerequisite to CIITA nuclear retention (Tosi et al., 2011). Nevertheless, the inability of CIITA to interact with Tax *in vitro* cannot be ascribed to the incapacity of CIITA to self-associate *in vitro*, because, as mentioned above, the dimerization-deficient CIITAΔ253–410 mutant retains the ability to inhibit Tax-1 *in vivo*. Other modifications of CIITA, including acetylation, deacetylation, and ubiquitination (Spilianakis et al., 2000; Wu et al., 2009 and references therein), might have a major role in Tax-1-binding. Such PTMs have been reported to affect the interaction of CIITA with cellular factors involved in MHC-II transcription (Greer et al., 2003) and the recruitment of either corepressors or coactivators on different promoters (Xu et al., 2008; Wu et al., 2009). PTMs-defective forms of both Tax and CIITA will be crucial to determine the potential role of specific modification in Tax-binding and/or functional inhibition. For instance, we have evidence that Tax-1 interacts with both the hypo- and the hyper-phosphorylated forms of CIITA (data not shown).

**FIGURE 3:**
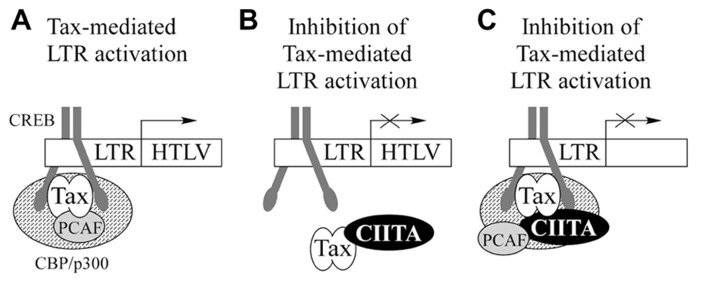
**CIITA-Tax interaction might differently affectTax-mediated activation of the viral LTR promoter.** **(A)** In the absence of CIITA, Tax-1 is recruited by CREB to the viral LTR and promote the formation of an higher order multiprotein complex activating transcription. **(B)** CIITA binds Tax and prevents its recruitment to the LTR promoter. **(C)** Tax bound to CIITA is recruited to the LTR, but its transcription function is impaired.

Overall, our findings suggest that CIITA-mediated inhibition of Tax activity could rely on the physical association between the two factors. We know that this binding occurs off DNA, but it is still unclear whether Tax–CIITA complexes are recruited on the HTLV LTR (**Figure 3A**). If this were case, two hypotheses are equally plausible. Tax bound to CIITA is not assembled on the viral promoter and this correlates with the inhibition of Tax-mediated LTR activation (**Figure 3B**). Alternatively, the binding of CIITA to Tax could still permit its recruitment on the LTR, but not its transcription function. CIITA might prevent the interaction of Tax with components of the transcriptional machinery required for HTLV LTR transactivation (**Figure 3C**). In this context, it is intriguing that, as discussed below, the direct interaction of Tax-1 with PCAF (P300/CBP-associated factor), which cooperates with Tax-1 to activate transcription from the LTR (Jiang et al., 1999), is severely impaired in the presence of CIITA (Tosi et al., 2011). To discriminate between the two above hypotheses, ChIP/EMSA assays are required. However, our subcellular distribution studies of Tax proteins in the presence of CIITA seem to favor the first mechanism. Tax-2 colocalizes with CIITA in the cytoplasm with a characteristic accumulation around the nuclear membrane potentially contributing to Tax-2 loss of function. Interestingly, Tax-2 does not respond by itself to LMB treatment (Chevalier et al., 2005); however, in cells treated with LMB, CIITA recruits Tax-2 into the nucleus (Orlandi et al., 2011). This suggests that CIITA exerts a driving force on Tax-2 distribution. More recently, extending these studies to Tax-1, we have shown that untagged Tax-1 expressed in 293T cells is trapped by CIITA in the cytoplasm of the majority of cells (Tosi et al., manuscript in preparation). Similarly to Tax-2, the impaired shuttling of Tax-1 into the nucleus, may account for the functional inhibition of Tax-1 by CIITA.

## Tax AND CIITA USE COMMON CELLULAR FACTORS TO CONTROL TRANSCRIPTION OF THEIR TARGET PROMOTERS

Tax interacts with a multitude of cellular factors, forming the so called Tax interactome, to modulate the expression of viral and host genes (Boxus et al., 2008; Simonis et al., 2012). Most of these physical and functional interactions derive from studies on HTLV-1 Tax-1. Much less is known about the cellular partners mediating Tax-2 biological functions. Notably, many of these Tax-interacting cellular proteins are used also by CIITA to activate the transcription of MHC-II promoters. These commonly utilized factors include transcriptional modulators, chromatin modifying enzymes, basal transcription factors, and transcription elongation factors (**Table 1**).

**Table 1:** Physical and functional interaction shared by Tax and CIITA.

Proteins	Function	Reference
CREB	Transcriptional activators	Kwok et al. (1996), Tosi et al. (2011), Moreno et al. (1999), Lochamy et al. (2007), Zhu et al. (2000).
NF-YB		Pise-Masison et al. (1997), Tosi et al. (2011), Orlandi et al. (2011), Jabrane-Ferrat et al. (2003), Zhu et al. (2000), Masternak et al. (2000).
TFIID	Basal transcription factors	Caron et al. (1993), Fontes et al. (1999a).
CBP, p300	Chromatin remodeling factors	Harrod et al. (1998), 2000, Kashanchi et al. (1998), Tosi et al. (2006), 2011, Kretsovali et al. (1998), Fontes et al. (1999b).
PCAF		Jiang et al. (1999), Tosi et al. (2006), Tosi et al. (2011), Spilianakis et al. (2000).
HDAC1		Ego et al. (2002), Lemasson et al. (2004), Lu et al. (2004), Zika et al. (2003).
BRG1		Wu et al. (2004) Easley et al. (2010), Zhang et al. (2006), Mudhasani and Fontes (2002).
CARM1		Jeong et al. (2006), Zika et al. (2005).
P-TEFb	Transcription elongation factors	Zhou et al. (2006), Cho et al. (2007), Kanazawa et al. (2000).

In addition, Tax and CIITA share several functional features. Neither are classical DNA-binding transcription factors, but instead interact with a platform of DNA-bound proteins to be recruited to the target promoters. Among them, CREB plays a major role promoting the formation of a multiprotein complex on DNA required for the full transcriptional activation (Kwok et al., 1996; Moreno et al., 1999; Zhu et al., 2000; Lochamy et al., 2007). In particular, Tax interaction with CREB docked at CRE sites of the viral 21-bp repeats, stabilizes the formation of the ternary complex and recruits the coactivators CBP/p300 and PCAF (Harrod et al., 1998, 2000; Kashanchi et al., 1998; Jiang et al., 1999). Another transcription factor that interacts with both Tax and CIITA, is the B subunit of the NF-Y complex, which binds the inverted CCAAT sequence in the Y-box of MHC-II promoters (Pise-Masison et al., 1997; Masternak et al., 2000; Zhu et al., 2000; Jabrane-Ferrat et al., 2003). While many investigations had assessed the contribution of NF-Y to class II transcription, no data regarding the specific role of NF-Y in HTLV transcription were available until recently. We confirmed the association between NF-YB and Tax-1 and extended it to the Tax-2 transactivator, which binds both to transfected and endogenous NF-YB in 293T cells (Orlandi et al., 2011; Tosi et al., 2011). For the first time we have shown that the over-expression of NF-Y significantly inhibited Tax-2-driven, but not Tax-1-driven LTR transactivation (Tosi et al., 2006, 2011). The reasons for this discrepancy are presently unknown and require further investigation. These findings, however, do not conclusively address how endogenous NF-Y might be important for modulating Tax transactivation capacity. It must be stressed that NF-Y is an ubiquitous factor and, seemingly, its physiologic levels do not impair Tax-2 transcriptional activity, as demonstrated by our Tax-dependent gene reporter assays performed in 293T/COS cells and by HTLV-2 productive infection of RJ.2.2.5 cells which constitutively express NF-Y (Tosi et al., 2006, 2011; Orlandi et al., 2011). Nevertheless, it is possible that endogenous NF-Y does not allow maximal Tax-2 transactivation and only its inactivation in cells by the use of dominant-negative NF-Y vectors or by small interfering RNA (siRNA; Mantovani et al., 1994; Dolfini et al., 2009) will provide clear evidence of its negative role in LTR promoter activity.

Several families of proteins binding to the Y-box sequence have been previously identified (Li et al., 1992; Wolffe et al., 1992). Besides NF-YB, two other factors called YB-1 and C/EBPβ have been shown to oppositely regulate HTLV-1 expression. The former increases the basal LTR transcription (Kashanchi et al., 1994), the latter, instead, down-regulates Tax-1-mediated transactivation (Hivin et al., 2004). Thus, distinct family members might exploit alternative mechanisms to modulate HTLV-2 and/or HTLV-1 transcription.

Several chromatin modifying factors, such as Brahma-related gene 1 (BRG1) and histone acetyltransferases (HATs) are commonly used by Tax and CIITA (**Table 1**). While it is well established that the recruitment of HATs to the 21-bp repeats by Tax plays a critical role in transactivation, it is not clear whether the ATP-dependent chromatin remodeling factor BRG1, also participates in Tax-mediated transactivation. Controversial reports have been published in the past on Tax-1–BRG1 functional interplay. In one study BRG1 was shown to interact with Tax-1 physically and functionally and to enhance its capacity to transactivate LTR promoter (Wu et al., 2004; Easley et al., 2010). On the contrary, another report indicated that Tax-1-mediated transactivation does not require BRG1 (Zhang et al., 2006). Interestingly, BRG1 has a dual action on MHC-II gene expression: (i) it induces CIITA pIV promoter activation (Pattenden et al., 2002), (ii) it is recruited by CIITA on MHC-II promoters where, by altering DNA topology, facilitates the access of general transcription factors and coactivators leading to gene expression (Mudhasani and Fontes, 2002). Thus the role of BRG1 in the regulation of HTLV-1 replication in presence of CIITA certainly requires further investigation.

The HATs p300, CBP, and PCAF participate with CIITA to the formation of an active MHC-II enhanceosome (Kretsovali et al., 1998; Fontes et al., 1999b). Moreover, they also catalyze the acetylation of CIITA at two N-terminal lysine residues within a bipartite nuclear localization signal (NLS). Acetylation or inhibition of deacetylation by Trichostatin A leads to increased nuclear levels of CIITA and higher transactivation of class II genes (Spilianakis et al., 2000). Remarkably, CIITA also contains an HAT activity, which is required for IFN-γ-activated MHC-I and MHC-II expression (Raval et al., 2001). While the transcriptional activity of CIITA is linked to the recruitment of HATs on class II promoters, histone deacetylation correlates with transcriptional repression and is mediated by distinct histone deacetylase (HDAC) complexes. HDAC1/HDAC2 stably associated with the mSin3A corepressor bind to CIITA and inhibits its transactivating function through a disruption of MHC-II enhanceosome (Zika et al., 2003). As far as the involvement of HATs in Tax-mediated HTLV transcription, our studies revealed functional differences among the HAT family members interacting with Tax-1 and Tax-2. In particular, we confirmed that HTLV-1 and HTLV-2 gene transcription is synergistically enhanced by the interaction of CBP/p300 with both Tax-1 and Tax-2. In contrast, Tax-1 but not Tax-2 selectively uses PCAF to optimally transactivate HTLV-1 LTR (Tosi et al., 2006, 2011). This effect is independent from the enzymatic activity of PCAF, which might instead engage other coactivators (Jiang et al., 1999). A selective usage of HATs by the two viral transactivators has been previously demonstrated for the inhibition of p53 by Meertens et al. (2004b). The different requirement for PCAF between Tax-1 and Tax-2 implies that only Tax-1 might affect nuclear PCAF-containing complexes, potentially contributing to the pleiotropic de-regulated expression of cellular genes during T cell transformation. Of note, a reduced transactivation and a defective cellular transformation have been observed with Tax-1 mutants which poorly interact with PCAF (Smith and Green, 1990; Jiang et al., 1999). These observations further support the idea that the higher oncogenic potential of Tax-1 with respect to Tax-2 might be, at least in part, attributed to a peculiar utilization of HATs. Similarly to CIITA, Tax-1 interacts with several HDACs including HDAC1, HDAC3, and HDAC6 (Villanueva et al., 2006; Legros et al., 2011). HDAC1 binding negatively regulates the HTLV-1 gene expression (Ego et al., 2002; Lemasson et al., 2004). Nevertheless, Tax-1 has been shown to replace HDAC1 on LTR promoter allowing transcription initiation (Lu et al., 2004). Thus, both CIITA and Tax may act as a molecular switch, to modulate transcription by coordinating the function of both HATs and HDACs. Besides the roles for HATs and HDAC, more recently arginine-specific methylation of histones has emerged as a critical feature for both MHC-II and HTLV-1 transcriptional regulation. The coactivator-associated arginine methyltransferase 1 (CARM1) has been reported to interact and synergize with both CIITA and Tax-1 to optimally activate transcription of their target genes (Zika et al., 2005; Jeong et al., 2006). Aside from their interactions with specific DNA-bound factors and chromatin modifying proteins, Tax and CIITA bind also component of the general transcriptional machinery, such as TFIID, to direct transcription initiation (Caron et al., 1993; Fontes et al., 1999a). There is also evidence that both transcription factors recruit the P-TEFb to the target promoters by interacting with the cyclin T1 subunit (Kanazawa et al., 2000; Zhou et al., 2006; Cho et al., 2007).

Overall, the findings discussed above highlight the central role of both Tax and CIITA in transcription through the coordination of enhanceosome complex assembly and the control of transcription initiation and elongation.

## CIITA EXPLOITS DIFFERENT MOLECULAR MECHANISMS TO INHIBIT THE VIRAL TRANSACTIVATORS Tax-1 AND Tax-2

**FIGURE 4:**
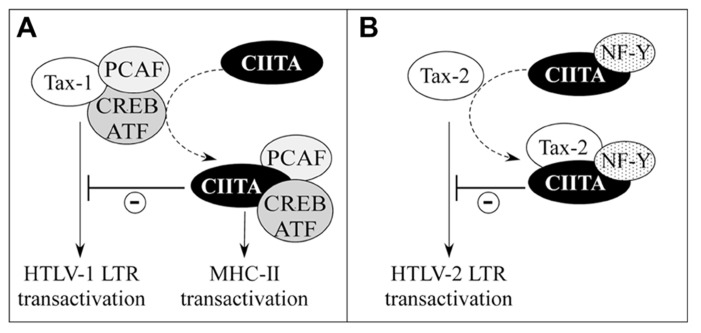
**Distinct mechanisms account for CIITA-mediated inhibition of HTLV-1Tax-1 and HTLV-2Tax-2.** **(A)** The cellular factors PCAF and CREB/ATF, required for CIITA-mediated MHC-II transcription, are sequestered by CIITA and are no longer available to interact physically and functionally with Tax-1. This results in the inhibition of HTLV-1 LTR transactivation (-). **(B)** CIITA facilitates the interaction between Tax-2 and NF-Y, an inhibitor of Tax-2 transcription function.

On the basis of what has been described in the previous section, the hypothesis that the physical interaction between CIITA and Tax may solely account for the observed CIITA-mediated inhibition of Tax-dependent LTR transactivation could reflect only part of the complex molecular interplay between the viral and cellular transactivators. Thus, we hypothesized that CIITA might inhibit Tax-mediated transcription also by sequestering one or more of those factors that are commonly used by CIITA and Tax for their specific transcriptional regulatory functions. This idea was further supported by our previous finding that CIITA inhibits HIV-1 replication by competing with Tat for the binding to cyclin T1 of P-TEFb complex (Accolla et al., 2002). Moreover, it is known that the sequestration of HATs is a common mechanism through which CIITA mediates gene suppression. For instance, CIITA by binding to and sequestering CBP inhibits metalloproteinase-9, collagen α 2(I), thymidine kinase, and cyclin D1 gene expression (Zhu and Ting, 2001; Nozell et al., 2004). In addition, CIITA by competing with NFAT (nuclear factor of activated T-cells) for p300 binding, represses the expression of IL-4 (Sisk et al., 2000). Similarly, CIITA exerts a repressive effect on Cathepsin E expression most likely via interaction with p300 (Yee et al., 2004). For these reasons we assessed whether the over-expression of some of these commonly used factors could rescue Tax function inhibited by CIITA. Whereas the over-expression of HATs did not overcome Tax-2 suppression, the over-expression of PCAF, but not of p300, counteracted the inhibitory function of CIITA on Tax-1 (Tosi et al., 2006, 2011). These data imply that CIITA might inhibit the recruitment of PCAF to the transcriptional complex on the viral promoter simply by sequestering it (**Figure 4A**). Nevertheless, another possibility is that the intrinsic ability of CIITA to interact with Tax-1, could impair Tax-1–PCAF association (**Figure 3C**). Indeed, CIITA decreased the *in vivo* binding of PCAF to Tax-1 (Tosi et al., 2011). CIITA and PCAF bind to two distinct regions of Tax-1 localized at the N-terminus and at the C-terminus, respectively (Jiang et al., 1999; Tosi et al., 2011) indicating that PCAF and CIITA do not compete for the same binding surface of Tax-1. Rather, the binding of CIITA to Tax-1 might alter the conformation of the viral transactivator masking the binding surface to PCAF.

Besides PCAF, the over-expression of CREB and ATF1 transcription factors restored CIITA-inhibited Tax-1 transactivation. Because the N-terminal region of Tax-1 interacting with CIITA includes CREB-binding site (Wu et al., 2004; Tosi et al., 2011), it is not surprising that the two factors compete for Tax-1. In the presence of CIITA, CREB might be no longer available for the recruitment of Tax-1 on the 21-bp repeats, thus preventing the assembly of the multiprotein complex required for optimal HTLV-1 LTR transactivation (**Figure 4A**).

Overall, these results indicate relevant differences in CIITA-mediated suppression of Tax-1 and Tax-2. The sequestration of HATs is not the major mechanism through which CIITA inhibits Tax-2, which, as discussed above, is functionally suppressed by NF-Y, another essential component of MHC-II enhanceosome. Interestingly, NF-Y cooperates with CIITA to inhibit Tax-2 transactivating function suggesting that CIITA might behave as a bridging factor to assemble a defective Tax-2/CIITA/NF-Y transcriptional complex (Tosi et al., 2006; **Figure 4B**). It has been reported that NF-Y interacts with PCAF (Currie, 1998) and it is intriguing that the two factors differently affect the transcriptional function of Tax-1 and Tax-2. Tax-1-mediated LTR transcription is enhanced by PCAF, but is not affected by NF-Y. On the contrary, Tax-2-mediated LTR transcription is inhibited by over-expressed NF-Y, but is not increased by PCAF. In this context CIITA seems to exploit the two players to exert its inhibitory function on the viral transactivators by inhibiting the interaction between Tax-1 and its positive coactivator PCAF, while increasing the binding of Tax-2 to its negative regulator NF-Y (Orlandi et al., 2011).

Future efforts will be devoted to assess whether CIITA inhibits Tax by sequestering other commonly used factors or by inhibiting their physical interaction with Tax-1 and/or Tax-2. At the present we can only exclude competition between CIITA and Tax for cyclin T1-binding. Indeed, the region of CIITA mediating Tat suppression differs from that required to inhibit both Tax-1 and Tax-2, indicating that CIITA blocks HIV-1 and HTLVs through different molecular mechanisms (data not shown).

## CONCLUSION

In this review we provided an update on the anti-viral features that make CIITA a fundamental link between adaptive and intrinsic immunity against HTLV infections. By inducing MHC-II expression and, thus, antigen presentation, CIITA triggers the activation of TH cells, which, in turn, orchestrate the adaptive immune responses against pathogens. In addition, CIITA has a direct inhibitory effect on the replication of HTLV-1 and HTLV-2 retroviruses by suppressing the transcriptional function of their viral transactivators. CIITA exploits different ways to exert this latter inhibitory function: it binds Tax-1 and Tax-2; it specifically modulates the interaction of Tax-1 and Tax-2 with relevant cellular factors; and it affects the subcellular localization of the two viral transactivators. Thus, CIITA seems to have evolved multiple strategies to be more effective in the inhibition of Tax function. It is known that other host restriction factors may target different steps of viral life cycle to block HIV-1 infection. Nevertheless, the restriction is more effective in species other than the human species, because HIV-1 has developed countermeasures against these innate defenses. In contrast, CIITA is a peculiar restriction factor because restricts HIV-1, HTLV-1, and HTLV-2 in their natural host (Accolla et al., 2002; Casoli et al., 2004; Tosi et al., 2011). So far, no viral products are known to counteract CIITA-mediated restriction. On the contrary, it is well known that some bacteria (e.g., Mycobacteria and Chlamydia) and some viruses (e.g., human cytomegalovirus and Varicella zoster) cause a reduction of MHC-II molecules on the surface of parasitized cells by inhibiting the pathway leading to the activation of CIITA gene transcription (Accolla et al., 2001). Thus, also HIV-1 and HTLV viruses might have evolved mechanisms to evade the host’s immune system based on the suppression of CIITA expression and/or function. At least for HIV-1 infection, our previous results indicated that Tat had no effect on both MHC-II and CIITA expression in T and macrophage cell lines (Tosi et al., 2000). As far as HTLV-1, it has been reported that Tax-1 increases basal MHC-II transcription by interacting with NF-YB (Pise-Masison et al., 1997). Moreover, a putative functional effect of Tax-1 on CIITA-mediated MHC-II gene expression, although unlikely, has not been investigated in detail as yet. Future investigation will thus uncover also the possible complementary role that both Tax-1 and Tax-2 might play a role in CIITA-dependent MHC-II transcription.

HTLV-1 and HTLV-2 viruses preferentially infect and replicate in human T lymphocytes, which express MHC-II molecules upon activation. It must be stressed, however, that whereas the expression of MHC-II molecules on the cell surface last for several days, CIITA expression is time-limited because of its very short half-life (Schnappauf et al., 2003). Thus, sustained expression of CIITA might be required to control HTLV replication. Interestingly, as discussed above, cell surface MHC-II molecule expression in mature DCs is up-regulated, whereas the expression of CIITA is silenced. This might, at least in part, explain why HTLV-1 productive infection is not counteracted in DCs (Jones et al., 2008). These findings disclose the opportunity of developing new therapeutic approaches against HTLV infections based on biological and/or pharmacological strategies aimed at up-regulating, in a controlled manner, the expression of CIITA in cells that are targeted by the virus.

Besides its role as transcriptional activator of HLTV genome transcription, Tax plays a major role in viral pathogenesis and T cell immortalization (Grassmann et al., 1989; Ross et al., 2000). Tax deregulates the expression of cellular genes mostly by the constitutive activation of NF-kB pathway and the inhibition of p53 tumor suppressor (Pise-Masison et al., 1998; Li and Gaynor, 2000; Mahieux et al., 2000; Miyazato et al., 2005; Peloponese et al., 2006; Qu and Xiao, 2011). It is conceivable that CIITA might exert a broader effect on HTLV infection by counteracting Tax oncogenic potential. Future efforts will be devoted to investigate whether CIITA inhibits Tax-mediated NF-kB activation and p53 suppression.

## Conflict of Interest Statement

The authors declare that the research was conducted in the absence of any commercial or financial relationships that could be construed as a potential conflict of interest.
